# Immunoglobulin G4-Related Spinal Intramedullary Inflammatory Pseudotumor: A Case Report and Literature Review

**DOI:** 10.3389/fneur.2022.878414

**Published:** 2022-06-21

**Authors:** Zhou Qi, Jianli Liu, Guoqiang Li, Yinian Zhang

**Affiliations:** ^1^Department of Neurosurgery, Lanzhou University Second Hospital, Lanzhou, China; ^2^Department of Medical Imaging, Lanzhou University Second Hospital, Lanzhou, China; ^3^Neurosurgery Center of Zhujiang Hospital of Southern Medical University, Guangzhou, China

**Keywords:** immunoglobulin G4-related disease, inflammatory pseudotumor, spinal intramedullary, imaging features, pathological features

## Abstract

Immunoglobulin G4-related disease (IgG4-RD) is an autoimmune disease that affects several organs. An inflammatory pseudotumor is a histologically proven benign tumor-like lesion that most commonly involves the lung and orbit. It is rare in the central nervous system, but rarest in the spinal canal. In this report, we present a case of IgG4-related intramedullary spinal inflammatory pseudotumor, along with a literature review. A 29-year-old male was transferred to the Department of Neurosurgery of Lanzhou University Second Hospital with progressive quadriparesis after numbness and weakness in both lower limbs for 50 days. Enhanced magnetic resonance imaging (MRI) of the spine revealed an isointense signal on T1-weighted images and a hyperintense signal on T2-weighted images from an enhanced mass located at the thoracic vertebrae region, for which a schwannoma was highly suspected. Then, a posterior median approach was adopted. The lesion was resected. The patient received further glucocorticoid after the diagnosis of an IgG4-related inflammatory pseudotumor was established, and the patient's symptoms improved, such as quadriparesis and lower limb weakness. This case highlights the importance of considering IgG4-related inflammatory pseudotumor as a differential diagnosis in patients with lesions involving the spinal intramedullary compartment and lower limb weakness when other more threatening causes have been excluded. IgG4-related inflammatory pseudotumor is etiologically unclear and prognostically unpredictable, and imaging may not help establish the diagnosis of IgG4-related inflammatory pseudotumor due to its resemblance to malignant tumors, and total resection might not be warranted. Glucocorticoid and surgery are usually the first-line treatments used.

## Introduction

Immunoglobulin G4-related disease (IgG4-RD) is a series of orphan autoimmune diseases that share key features and affect nearly all systems throughout the body (1). IgG4-RD is a fibrous-inflammatory disease associated with immunomodulation. The most commonly affected organs are the pancreas, bile duct, major salivary glands, lacrimal glands, retroperitoneum, and lymphatic ducts (2). In the central nervous system, IgG4RD has been described in the pituitary gland and the brain parenchyma. Furthermore, it is thought to be responsible for several cases of pachymeningitis. In the spine, the location of this type of lesion is mainly extramedullary subdural, and epidural space and intramedullary lesions have been rarely reported in the literature. To our knowledge, this is the first report of IgG4-related intramedullary spinal inflammatory pseudotumor.

## Case Presentation

### Medical History

A 29-year-old male presented with numbness, weakness, and tightness in both lower limbs (left followed by right) for the previous 50 days. He had not been able to stand and walk for 4 days. He had no history of fever, trauma, or night sweats but had a history of dysuria.

A neurological examination of the patient revealed weakness in both lower limbs, and the muscle strength of the left lower limb was grade 2, and that of the right lower limb was grade 3. Temperature sensation, pain, and touch decreased significantly at and below the umbilical plane. The patient could not complete the heel, knee, and tibia tests, or undergo an examination of the Romberg sign. Pathological signs were not observed. The patient had a soft neck with no resistance.

**Figure 1:**
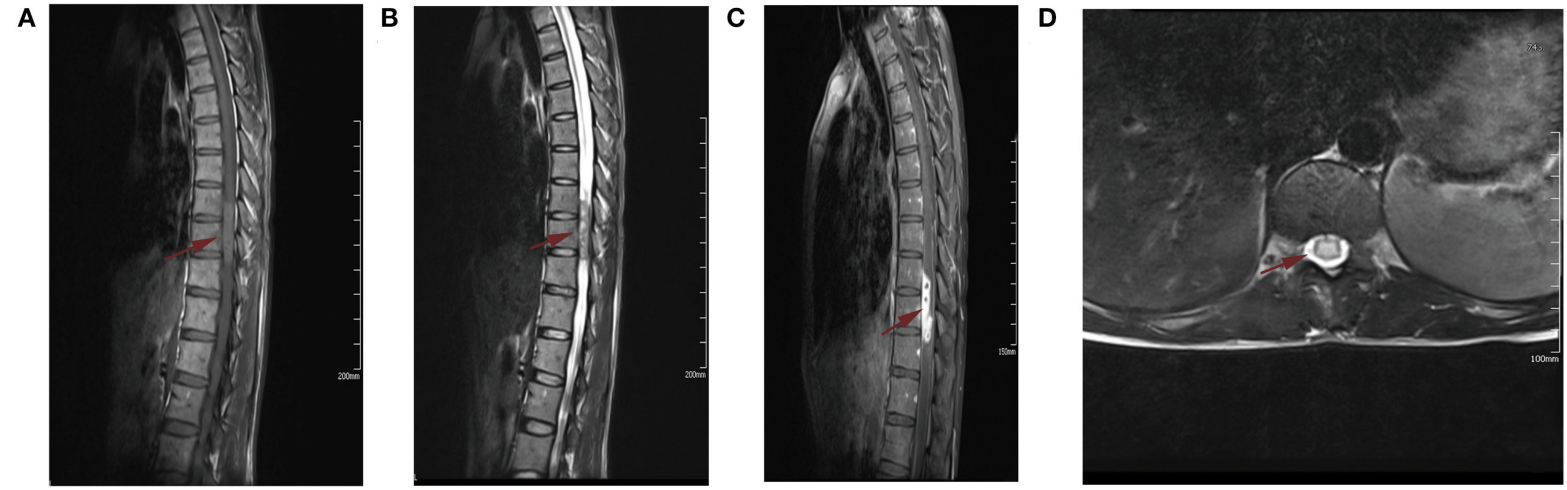
Plain and contrast enhance magnetic resonance imaging (MRI). **(A)** Sagittal T1-weighted MRI showed isointensive signal lesion in spinal cord at T9-10 level. **(B)** Sagittal T2-weighted MRI demonstrated a hyperintense signal mass. **(C)** Sagittal postcontrast T1-weighted MRI showed the mass was homogeneously enhanced. **(D)** Axial postcontrast T1-weighted MRI showed the lesion was locally connected to the nerve root. The Red arrow is mainly used to show the location of the lesion.

Magnetic resonance imaging (MRI) of the spine showed a subdural heterogeneous signal in the spinal canal at the level of the thoracic (3, 4) vertebrae, while slight hyperintensity on T2WI and multiple nodular cystic signals were also shown. An enhanced scan showed obvious heterogeneous enhancement, and no obvious enhancement of the cystic area was observed in the lesion. The lesion was locally connected and adhered to the nerve roots, which were thickened and showed enhancement. The central spinal canal of the upper and lower segments of the thoracic (5–7) and thoracic (8), lumbar (1) vertebral bodies were expanded, and the adjacent dura was thickened and enhanced. Moreover, the lower segment of the cauda equina was thickened and expanded (Figure 1). The erythrocyte sedimentation rate, serum C-reactive protein level, Monteux test, and M-band electrophoresis results were normal.

### Surgical Details

**Figure 2:**
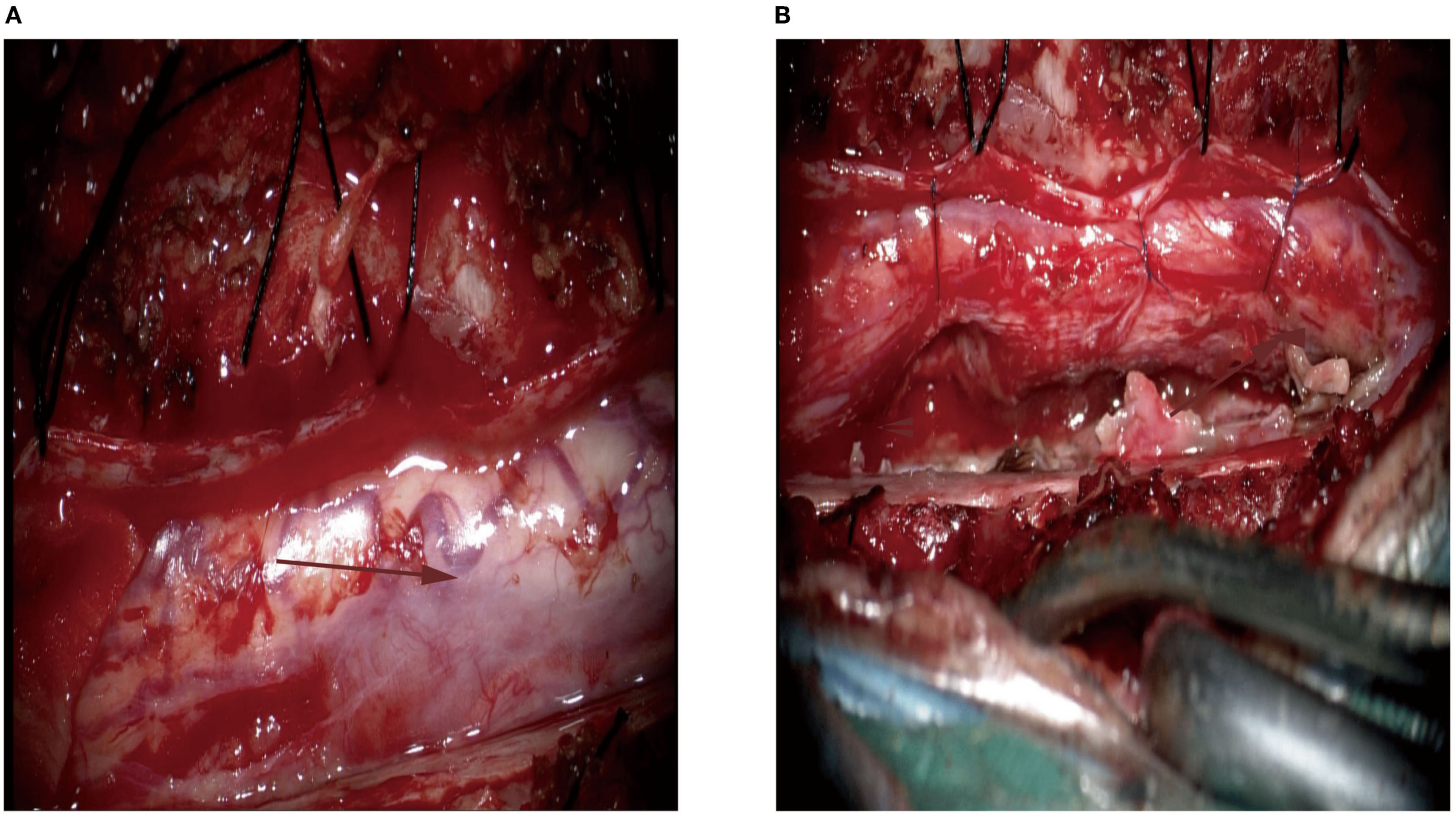
Intra-operative image of the lesion located at the right ventral margin. **(A)** The lesion had abundant blood supply (the arrow); **(B)** The gross total resection was achieved. The Red arrow is mainly used to show the location of the lesion.

After successful intubation under general anesthesia, the patient was placed in the prone position, and a straight incision was made in the middle of the T (3)–T (9) segment. Microscopically, we identified a 2.5-cm tumor in the right ventral margin, extending from the spinal cord to the external surface, and the tumor capsule was not intact. Therefore, the spinal cord and tumor boundary were unclear (Figure 2A). The tumor had an abundant blood supply, and the adjacent dura mater adhered to the tumor. Careful separation, protection of the spinal cord, and complete microscopic resection of the tumor were performed (Figure 2B). The mass was subjected to histopathology, immunohistochemistry, and infectious diseases workup.

### Pathological Examination

**Figure 3:**
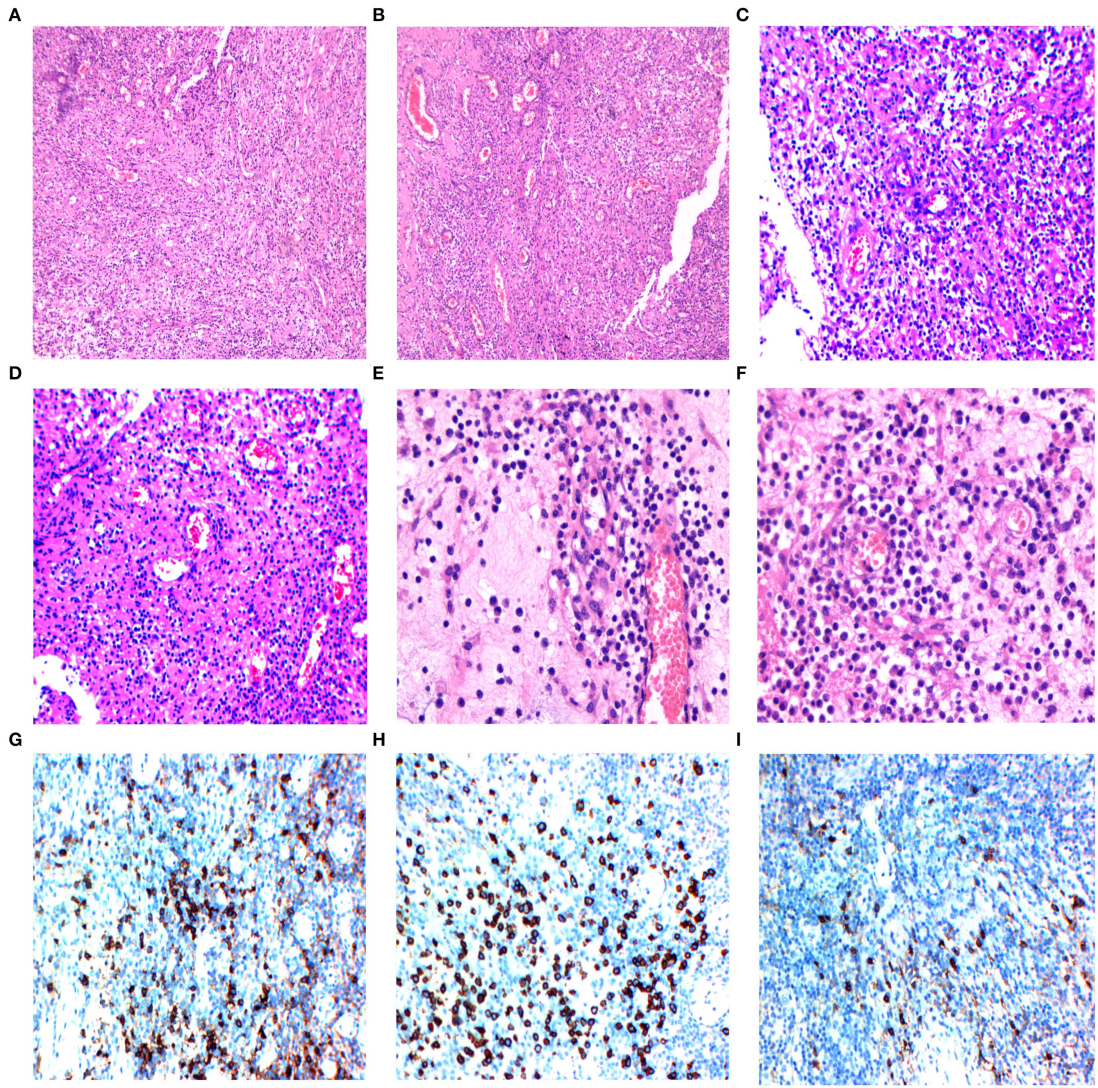
**(A,B)** The histology of the intramedullary lesion showed chronic inflammatory lymphoplasmacytic infiltrate with fibrosis, 10 × 10 magnified view showed inflammatory cell collections (Hematoxylin-eosin, magnification 100×). **(C,D)** 20 × 10 magnified view demonstrated chronic inflammatory infiltration (hematoxylin-eosin, magnification 200×). **(E,F)** 40 × 10 magnified view showed rich lymphoplasmacytic infiltrates (plasma cells, eosinophils, macrophages, and lymphocytes) with storiform arrangement of spindle cells and dense fibrosis. **(G)** Immunohistochemistry with plasma cells (CD38+) in the infiltration area. **(H)** Immunohistochemistry with plasma cells (CD138+) in the infiltration area. **(I)** The IgG4+ cells account for more than 50% of the plasma cells.

The sample consisted of dense fibrous and meningeal tissue, with polypoid changes on the surface. Infiltration of the spinal cord parenchyma was observed under a microscope. Histopathological examination revealed multiple fragments composed of round cells and patches of oval-to-spindle cells. The population of inflammatory cells is mainly composed of lymphocytes, with a small proportion of histiocytes, neutrophils, eosinophils, and plasma cells. Our differential diagnoses included lymphoma, meningioma, neurilemmoma, and small cell malignancies (Figures 3A–F).

### Immunohistochemical Staining

The tumor cells showed positive vimentin, MPO, CD3, CD20 staining, negative cytokeratin pan (CKp), S-100, GFAP, synaptophysin (Syn), NeuN, Olig-2, INI-1, CD117 staining, and the Ki-67 proliferation index were 20% on immunohistochemical staining. The positive immunoglobulin G4 staining of plasma cells showed gross cellularity of >50/high-power field (Figure 3I) and positive CD138 staining (Figure 3H). The histiocytes showed positive CD68 staining (Figure 3G). In addition, the intraoperative tumor morphology was atypical, making the differential diagnosis challenging. Blood samples for further immune disease investigations demonstrated no elevated levels of IgG in the serum, however, the histological IgG4/IgG ratio was 54% (>40%). The diagnosis of IgG4-related spinal intramedullary inflammatory pseudotumor was established by a multidisciplinary team (MDT).

The patient recovered well-post-operatively. 2 days after the operation, the recovery of deep and shallow sensations below the umbilical plane began to occur. After 14 days, the muscle strength of the left lower limb was restored, the muscle strength of the right lower limb was 4/5 (Medical Research Council grading), and the patient's motor function recovered as well. At the second follow-up 3 months after the surgical procedure, the patient was found to be able to perform normal activities.

## Discussion

To our knowledge, this is the first case of IgG4-associated inflammatory pseudotumor extending from the intramedullary to the extramedullary compartment. IgG4-RD is a rare autoimmune disorder that mimics malignancy. IgG4-RD predominantly manifests in the pituitary gland, brain parenchyma, meninges, and peripheral nerves and rarely in the epidural tissue of the central nervous system (3). Spinal IPT in an extrapulmonary location is very rare, with it being most common in the cervical spine and occurring less frequently in the thoracic or thoracolumbar spine region (4). At the same time, they are predominantly located intradural extramedullary and rarely extradural or intramedullary. Recently, seven cases of IgG4-RD IPT have been reported (Table 1). However, no case of intramedullary IPT was identified in the literature. This case is unique in that the imaging and intraoperative findings are highly suspicious of neurilemmoma and primitive neuroectodermal tumors. Therefore, IPT should be considered in the clinical and imaging differential diagnoses.

**Table 1:** Recently reported cases of spinal Ig4 intramedullary inflammatory pseudotumor in the literature.

**Report**	**Age(year/sex)**	**Publication time**	**First symptoms**	**Serum IgG4 level**	**Treatment**	**Location**	**Bony involvement**	**MRI**			**References**
								**T1**	**T2**	**T1 Contrast**	
Abdulla et al.	27/M	2021	Weakness in both lower limb	Normal	Surgery and steroid treatment	T5-T6	No	Iso	Hypo-Hyper	+	(5)
Winkel et al.	48/F	2018	Lower back pain	Normal	Surgery and steroid treatment	L2-L3	No	Iso	NR	+	(6)
Bridges et al.	68/M	2019	Neck radiation pain	NR	Surgery and steroid treatment	T4-T5	No	Hypo	Hypo-Hyper	+	(7)
Rumalla et al.	50/M	2017	Severe back pain	Normal	Biopsy positivity staining steroids positivity staining antibiotics treatment	T5-T6	Yes	Iso	NR	+	(8)
Williams et al.	46/F	2017	Pain and weakness in the left upper extremity	Normal	Surgery and steroid treatment	C4-T1	No	Iso	NR	+	(9)
Merza et al.	60/F	2019	Weakness and numbness in all four extremities	Rise	Surgery and steroid treatment	T4-T5	No	Iso	Hypo-Hyper	+	(10)
Ferreira et al.	57/M	2016	Severe paraparesis, gait disturbance	Normal	Surgery and steroid treatment	T2-T12	No	Iso	Hypo-Hyper	+	(11)

*MRI, magnetic resonance imaging; F, female; M, male; Iso, iso intensity; Hypo, hypo intensity; NR, not reported; Hyper, hyper intensity*.

The research by Ferreira et al. (11) showed that plasmacytosis was first detected in biopsy of inflammatory pseudotumor, with more than 50% IgG4-positive plasma cells in the thoracic spine. We used the PubMed database to identify seven cases (2016–2020) of IgG4-RD IPT (5–11). In these cases, the mean age of the patients was 44.5 years, with the majority being male. Moreover, the initial symptoms were mainly lower back pain, numbness, and weakness in the lower limbs. Four patients progressed to paresis without any special symptoms. Only one case had bony involvement and an increased level of serum IgG4, demonstrating that the increase in serum IgG4 cannot be used as a necessary condition for diagnosing IgG4-RD IPT. All lesions showed distinct contrast enhancement on the MRI scan, and the T1-weighted images were mainly isointense. The radiologic features of IPT were similar to those of spinal epidural lymphomas, meningiomas, metastasis, and plasmacytomas, except for intact adjacent bone and intensive and homogenous contrast enhancement without a Dural tail sign (12).

In summary, clinical findings, radiological features, and serum IgG4 concentrations are not sensitive or specific for the diagnosis of IgG4-related IPT, making its diagnosis particularly difficult. Furthermore, many other diagnoses must be eliminated before IgG4-related IPT is suspected. Therefore, attention should be paid to the major histopathological features of IgG4-RD, including lymphoplasmacytic infiltrate, fibrosis, and obliterative phlebitis (13). An elevated histologic IgG4/IgG ratio (>40%) may not be necessary for diagnosing IgG4-related IPT. Nevertheless, we recommend the use of IgG4/IgG ratio to better support the diagnosis. Surgical procedures combined with steroids are considered an appropriate treatment strategy.

## Conclusion

The intramedullary lesion may represent inflammatory pseudotumor. When more common conditions such as malignant tumors, infectious causes, or schwannomas are excluded, IgG4-RD should also be considered in the differential diagnosis of spinal inflammatory mass. This case report and literature review highlight another potential diagnosis in the setting of intramedullary space-occupying lesions.

## Data Availability Statement

The original contributions presented in the study are included in the article/Supplementary Material, further inquiries can be directed to the corresponding author/s.

## Ethics Statement

The studies involving human participants were reviewed and approved by Research Ethics Committee of Lanzhou University Second Hospital. The patients/participants provided their written informed consent to participate in this study.

## Author Contributions

ZQ, JL, GL, and YZ were involved in patient treatment, data collection and analysis, and manuscript writing. All authors contributed to the article and approved the submitted version.

## Funding

Basic Research Innovation Group Project of Gansu Province (21JR7RA432) and Cuiying Scientific and Technological Innovation Program of Lanzhou University Second Hospital (CY2021-MS-B11).

## Conflict of Interest

The authors declare that the research was conducted in the absence of any commercial or financial relationships that could be construed as a potential conflict of interest.

## Publisher's Note

All claims expressed in this article are solely those of the authors and do not necessarily represent those of their affiliated organizations, or those of the publisher, the editors and the reviewers. Any product that may be evaluated in this article, or claim that may be made by its manufacturer, is not guaranteed or endorsed by the publisher.
