# Supplementary figures and images for: Immunoglobulin G4-Related Spinal Intramedullary Inflammatory Pseudotumor: A Case Report and Literature Review
Source: Front Neurol. 2022 Jun 21;13:878414. doi: 10.3389/fneur.2022.878414 (PMC9275449; doi:10.3389/fneur.2022.878414)

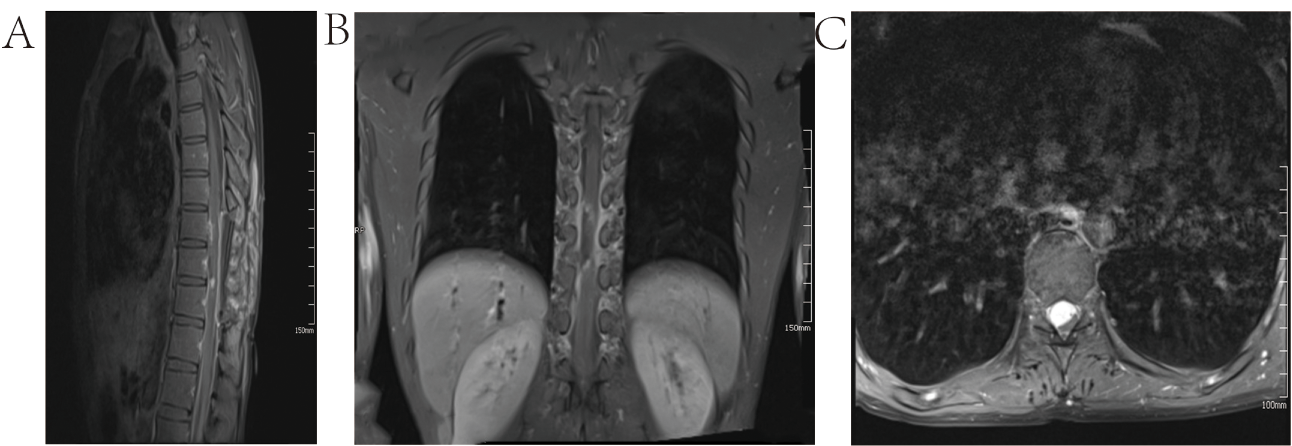

Supplement: Supplementary file 1 [file Image_1.TIF]

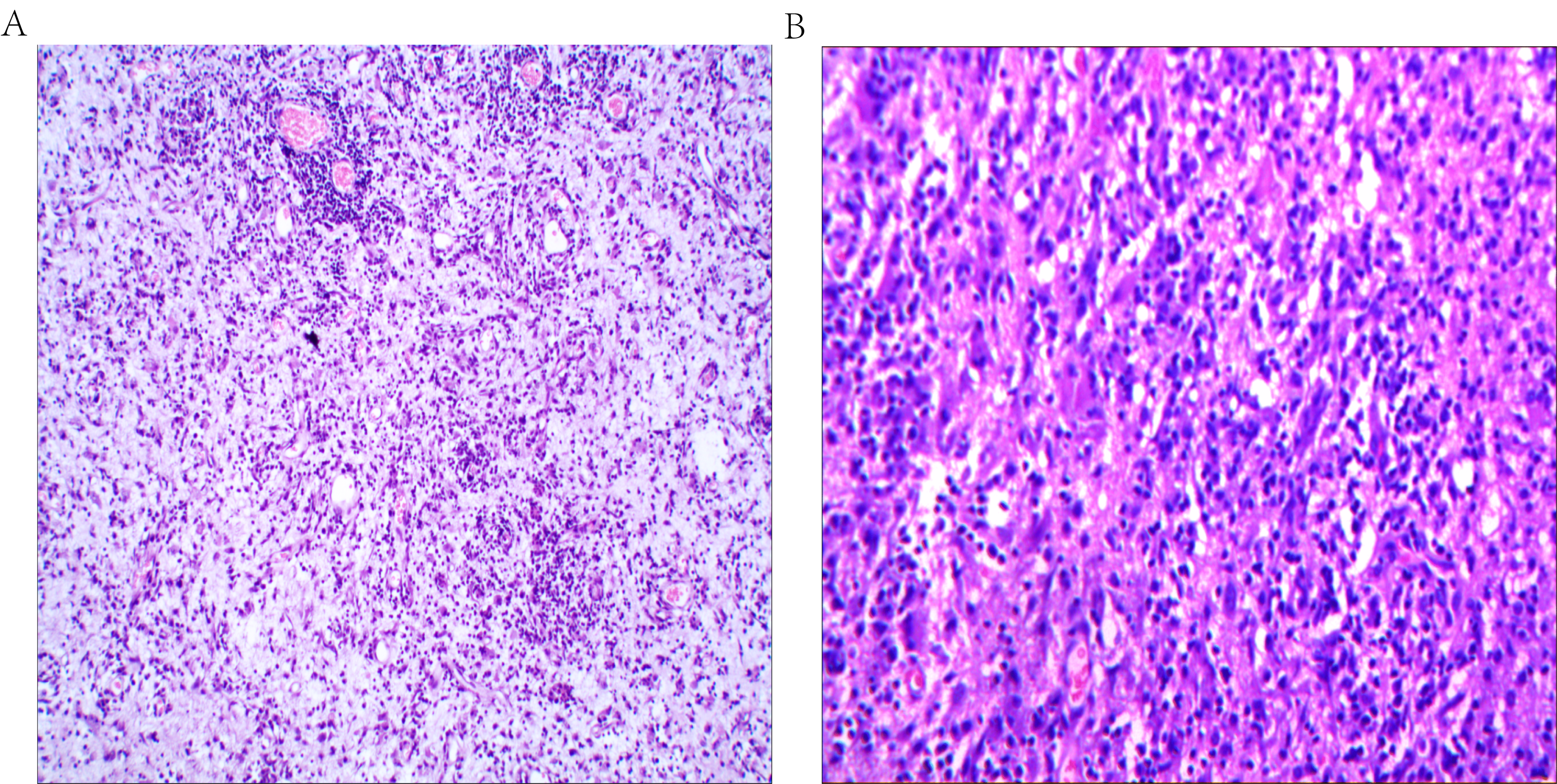

Supplement: Supplementary file 2 [file Image_2.TIF]
